# Same day HIV diagnosis and antiretroviral therapy initiation affects retention in Option B+ prevention of mother-to-child transmission services at antenatal care in Zomba District, Malawi

**DOI:** 10.7448/IAS.19.1.20672

**Published:** 2016-03-11

**Authors:** Adrienne K Chan, Emmanuel Kanike, Richard Bedell, Isabel Mayuni, Ruth Manyera, William Mlotha, Anthony D Harries, Joep J van Oosterhout, Monique van Lettow

**Affiliations:** 1 Dignitas International, Zomba, Malawi; 2 Division of Infectious Diseases, Sunnybrook Health Sciences Centre, University of Toronto, Toronto, Canada; 3 Dalla Lana School of Public Health, University of Toronto, Toronto, Canada; 4 Zomba District Health Office, Malawi Ministry of Health, Zomba, Malawi; 5 International Union Against Tuberculosis and Lung Disease, Paris, France; 6 London School of Hygiene and Tropical Medicine, London, UK; 7 Department of Medicine, University of Malawi College of Medicine, Blantyre, Malawi

**Keywords:** PMTCT, Option B+, Malawi, service delivery model, ANC–ART integration, retention on ART

## Abstract

**Introduction:**

Data from the Option B+ prevention of mother-to-child transmission (PMTCT) program in Malawi show considerable variation between health facilities in retention on antiretroviral therapy (ART). In a programmatic setting, we studied whether the “model of care,” based on the degree of integration of antenatal care (ANC), HIV testing and counselling (HTC) and ART service provision–influenced uptake of and retention on ART.

**Methods:**

We conducted a retrospective cohort study of pregnant women seeking ANC at rural primary health facilities in Zomba District, Malawi. Data were extracted from standardized national ANC registers, ART registers and ART master cards. The “model of care” of Option B+ service delivery was determined at each health facility, based on the degree of integration of ANC, HTC and ART. Full integration (Model 1) of HTC and ART initiation at ANC was compared with integration of HTC only into ANC services (Model 2) with subsequent referral to an existing ART clinic for treatment initiation.

**Results and discussion:**

A total of 10,528 women were newly registered at ANC between October 2011 and March 2012 in 23 rural health facilities (12 were Model 1 and 11 Model 2). HIV status was ascertained in 8,572 (81%) women. Among 914/8,572 (9%) HIV-positive women enrolling at ANC, 101/914 (11%) were already on ART; of those not on treatment, 456/813 (56%) were started on ART. There was significantly higher ART uptake in Model 1 compared with Model 2 sites (63% vs. 51%; *p*=0.001), but significantly lower ART retention in Model 1 compared with Model 2 sites (79% vs. 87%; *p*=0.02). Multivariable analysis showed that initiation of ART on the same day as HIV diagnosis, but not model of care, was independently associated with reduced retention in the first six months (adjusted odds ratio 2.27; 95% CI: 1.34–3.85; *p*=0.002).

**Conclusions:**

HIV diagnosis and treatment on the same day was associated with reduced retention on ART, independent of the level of PMTCT service integration at ANC.

## Introduction

Malawi conceived the Option B+ PMTCT strategy and started scaling up its coverage in 2011 [1,2]. Despite limited availability of information on the effectiveness and impact of population-level implementation, this program has been rapidly adopted in other sub-Saharan African countries with generalized epidemics, largely for programmatic reasons.

Existing early literature has shown that barriers to successful implementation throughout the cascade of Option B+ services may affect the uptake and adherence to ART by pregnant women, the follow-up of HIV-exposed infants and the long-term retention of this patient population [3,4]. Considerable variation in retention on ART of Option B+ women exists between health facilities [4]. Urgent research priorities at the national level with the Malawi Ministry of Health (MOH) have been identified that include evaluation of barriers and facilitators to uptake of lifelong ART and long-term retention by largely asymptomatic and newly diagnosed HIV-positive women seeking antenatal care (ANC) [5].

From October 2011 onwards, Malawian health facilities rolled out Option B+ as part of routine service delivery. However, the new policy was not prescriptive as to how to organize the integration of ANC, HIV testing and counselling (HTC) and ART services nor was any existing evidence available. Health facilities therefore made decisions about the organization of PMTCT care on the basis of factors related to their specific circumstances. A previous study describing and comparing Option B+ service delivery models in six of Malawi's twenty-eight districts using facility-level MOH cohort reports demonstrated large variability in how services had been integrated and observed associations between the organization of service delivery and both uptake of HIV testing and retention in care on ART [6].

Our aim was to evaluate early programmatic outcomes among newly diagnosed HIV-positive women who presented to rural primary health facilities for ANC services. Specifically, we assessed the impact of integration of HIV testing and ART at ANC clinics and the initiation of ART on the same day as HIV diagnosis on uptake and retention on ART.

## Methods

Services for both ANC and ART were provided at 30 health facilities in Zomba District (population 670,000 [7]; HIV seroprevalence 16.5% [8]) in Southern Malawi. Urban health facilities and referral centres were excluded from the study, in the interest of gaining a clearer understanding of the primary care experience. Twenty-three primary health facilities were included in the study (21 rural, 2 peri-urban).

Using national guidelines, treatment protocols and monitoring and evaluation (M and E) tools [7], decentralized Option B+ services were coordinated by the Zomba District Health Office with the support of Dignitas International, a Canadian medical research and humanitarian organization that has supported HIV service provision in South East Malawi since 2004 [8]. Guidelines allow ART initiation on the same day as HIV diagnosis, after a single individual counselling session, with optional deferral of the recommended group counselling session to the first follow-up visit.

We conducted a retrospective cohort study of all consecutive pregnant women seeking ANC at 23 rural primary health facilities in Zomba District, Malawi, between 1 October 2011 and 31 March 2012 and collected data from standardized Malawi MOH monitoring and evaluation tools. At the time of study, all health facilities kept individual patient-level data on ANC service provision in ANC registers, and individual patient-level data on ART service provision in standardized ART master cards and ART registers. Facility-level data were aggregated at all health facilities every three months and entered into an electronic database for cohort reporting by the MOH Department of HIV and AIDS M&E team. Patient-level data on ANC and ART services were not linked as part of the existing M&E system.

We collected health facility-level data on the number of registrations, HIV testing, new HIV-positive diagnoses and HIV-positive women already on ART from ANC registers. In order to further determine true uptake of ART, for every patient who had documentation of ART registration in the ANC register, a trained research assistant searched for corresponding evidence of registration in the facility ART register using linkage either by ART number or patient name (and matching age for confirmation). To best optimize determination of primary patient outcomes, the research assistant extracted individual patient outcome data from corresponding ART master cards. Only data from patients whose ART master cards could be found were included, as these outcomes are not recorded in ANC registers and were irregularly updated in ART registers. Lost to follow up was defined as not attending clinic greater than two months after the date that last dispensed antiretroviral drugs would run out. We described the Option B+ service delivery “model of care” in terms of the degree of integration of ANC, HTC and ART. Full integration (Model 1) of HTC and ART initiation at ANC was compared with integration of HTC only into ANC services (Model 2) with subsequent referral to an existing ART clinic for treatment initiation. The model of care used at a health facility was determined by PMTCT and ART Program Coordinators and Clinical Mentors who visit each health facility regularly and confirmed by the Zomba District Health Office.

Data analyses were conducted with IBM SPSS Statistics 20 (IBM, Armonk, NY, USA). Facility- and patient-level data were described with proportions and 95% confidence intervals (CI) or medians with interquartile ranges (IQR) by site and by model of ANC–ART integration.

Facility-level data included number of women (newly) registered and tested for HIV, number of previous and new HIV positives and number of HIV positives already on ART. Patient-level data included timing and uptake of and retention on ART at three and six months from initiation. As per the national guideline, women on ART were counted as “lost to follow up” if they were expected to have run out of ART for two or more months (based on the number of tablets given at the last visit) and were not known to have transferred out, stopped or died. Comparisons between models were made using chi-square tests and nonparametric independent sample median tests.

In order to consider a cascade of Option B+ services at each facility, we extrapolated *all women who were projected to be eligible for Option B+ ART* to be defined as follows: (HIV prevalence observed in model x the number not tested in model) + new HIV positives in model.

To explore factors associated with being lost to follow up within the first six months from starting ART, binary logistic regression models were fitted with being lost to follow up by six months as the outcome variable. Crude odds ratios (OR) and adjusted odds ratios (aORs) with 95% CI's were calculated and were controlled for all available variables in the model, namely, mothers’ age, parity, health facility model of care, health facility monthly number of ANC registrations and timing of ART initiation. A significance level of 0.05 was set for all statistical testing. Ethics approval was obtained from the Malawi National Health Science Research Committee (#1084) and the International Union Against Tuberculosis and Lung Disease Ethics Advisory Group (#71/12). As this was a retrospective record review, no consent was required.

## Results

A total of 10,528 pregnant women were registered at ANC clinics during the study period, with 3,842 registered at Model 1 (*n*=12) clinics and 6,686 at Model 2 (*n*=11) clinics (Table 1).

**Table 1:** Women registered and tested for HIV at ANC, timing and uptake ART stratified by model of ANC–ART integration

	ALL sites	Model 1	Model 2
Number of sites	23	12	11
**1. Facility-level data**			
Total no. of women registered at ANC	10,528	3,842	6,686
New ANC registrations/month/site, median (IQR)	54 (33–119)	44 (20–65)	100 (52–140)
Total HIV testing done (% of women registered at ANC)	8,572 (81%)	3,379 (88%)	5,193 (78%)
Total previous HIV positives	288 (3%)	110 (3%)	178 (3%)
Total new HIV positives	626 (6%)	273 (7%)	353 (5%)
All HIV-positive women (% of women registered at ANC)	914 (9%)	383 (10%)	531 (8%)
HIV positives already on ART (% of previous HIV positive)	101 (35%)	39 (36%)	62 (35%)
**2. Individual patient-level data**			
Total nr of women needing to start ART	813	344	469
Total started ART (% ART master cards found of women needing to start ART)	456 (56%)	217 (63%)	239 (51%)
Total started ART on same day as HIV diagnosis	170 (37%)	124 (57%)	46 (19%)

**Model 1:** HIV diagnoses and ART initiation at ANC. **Model 2:** HIV diagnoses at ANC, but referral to ART for ART initiation.

In individual patient-level data analysis of *the women who were found to be HIV positive and eligible for ART*, there was a significantly higher rate of ART uptake in Model 1 compared with Model 2 sites (63% vs. 51%; p 0.001). The median number of days between HIV diagnosis and ART start was 0 (IQR 0–28) in Model 1 sites and 26 (IQR 4–88) in Model 2 sites (*p*=0.001). In Model 1 sites, 57% of women started ART on the same day as HIV diagnosis versus 19% (*p*=0.001) in Model 2 sites.

Among *HIV-positive women who actually started ART*, there was a significantly higher loss-to-follow-up rate at six months in those initiating in Model 1 compared with Model 2 sites (22% vs. 8%; *p*=0.001). There was significantly higher retention at three and six months in Model 2 sites compared with Model 1 sites (Table 2).

**Table 2:** Three and six months ART treatment outcomes, stratified by model of ANC/ART integration

*Treatment outcomes among women who started ART*				

	ALL sites	Model 1	Model 2	*p*
Cumulatively retained on ART at three months from initiation	381 (84%)	172 (79%)	209 (87%)	0.02
Cumulatively retained on ART at six months from initiation	368 (81%)	162 (75%)	206 (86%)	0.003
Lost to follow up at six months	66 (15%)	47 (22%)	19 (8%)	0.001
Transferred out at six months	13 (3%)	5 (2%)	8 (3%)	0.58
Stopped ART at six months	7 (2%)	3 (1%)	4 (2%)	0.58
Died at six months	2 (0.5%)	0	2 (1%)	0.5

**Figure 1:**
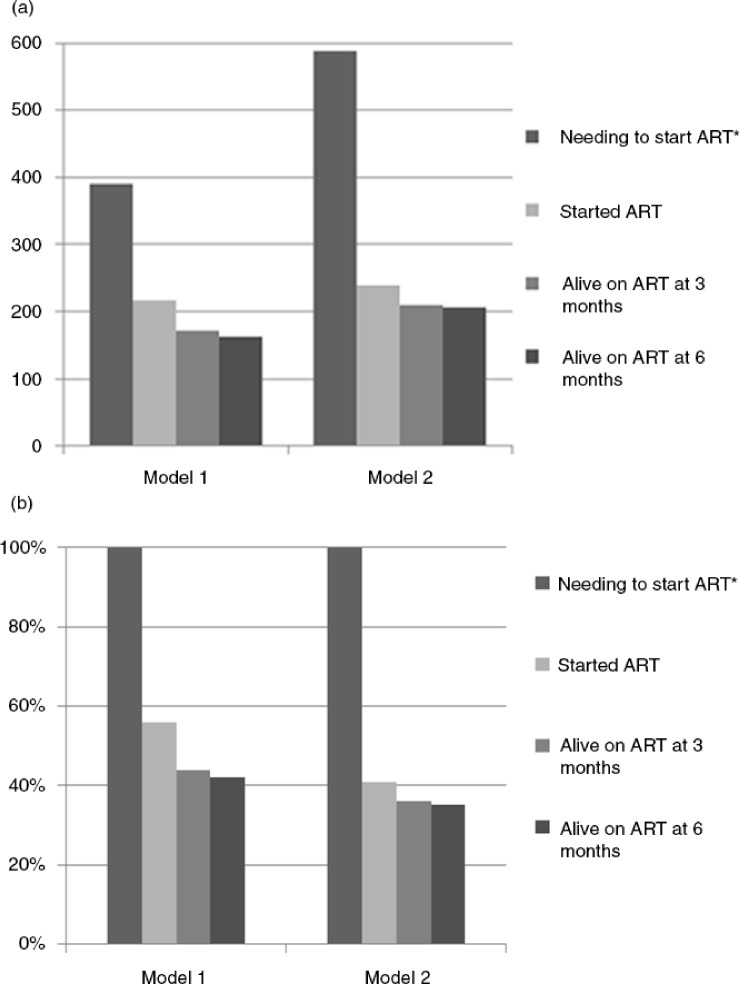
(a) Number and (b) proportion of women starting and retaining in ART care when extrapolating number of women eligible for Option B+ ART in cascade of care. *Number of women projected to be eligible for Option B+ ART=(HIV prevalence observed in model x the number not tested in model) + new HIV positives in model.

To model a cascade of Option B+ services at health facilities by “model of care,” we then considered *all women who were projected to be eligible for Option B+ ART* in Figure 1, combining facility-level and individual patient-level data. Because point estimates of HIV testing and of ART initiation were higher in Model 1 than in Model 2 facilities, the modelled percentages of ART eligible women who were retained on ART at three and six months were higher in Model 1 facilities (Figure 1a and b).

Multivariable analysis (Table 3) showed that only initiation of ART on the same day as HIV diagnosis was independently associated with reduced retention in the first six months (aOR 2.27; 95% CI: 1.34–3.85; *p*=0.002).

**Table 3:** Factors associated with being lost to follow up within the first six months from starting ART

Characteristics	*n*	Crude (95% CI)	*p*	Adjusted OR* (95% CI)	*p*
Mothers’ age (in years)	456	0.98 (0.94–1.02)	0.25	0.97 (0.92–1.02)	0.19
Parity	456	0.99 (0.89–1.12)	0.99	1.00 (0.89–1.14)	0.96
Size of health facility (ANC registrations/month)	46	1.21 (0.56–2.62)	0.63	1.20 (0.53–2.69)	0.66
<33	169	1.10 (0.66–1.80)	0.72	1.47 (0.86–2.51)	0.16
IQR 33–119 > 119	241	–	–	–	–
ART initiation at ANC (Model 1)	217	0.49 (0.30–0.79	0.003	1.68 (0.98–2.88)	0.058
ART initiation at ART clinic (Model 2)	239	–	–	–	–
HIV diagnosis and ART initiation					
On Same day	170	2.55 (1.58–4.13)	0.001	2.27 (1.34–3.85)	0.002
At least 1 day between HIV diagnosis and ART initiation	277	–	–	–	–

* Adjusted for all variables in the models.

## Discussion

In this study of mainly rural health centres in Malawi, after the introduction of Option B+, uptake of ART was higher in health facilities where ANC clinics provided integrated HTC and ART initiation services (Model 1) compared with health facilities where ANC clinics only provide HTC and ART was initiated outside the ANC clinic (Model 2). However, pregnant women in Model 1 clinics had more than twice the risk of defaulting from care at six months. Defaulting was independently associated with starting ART on the same day as the HIV diagnosis. Losses from care were small between three and six months after ART initiation across both models. The negative effect of Model 1 on defaulting may be mitigated by a higher uptake of testing and ART initiation. This was suggested by a cascade of care projection showing that of women needing to start ART for PMTCT, the percentage remaining in care after six months may be higher in Model 1 clinics. The higher HIV testing coverage in Model 1 clinics has a large impact on this projection.

Early studies after the introduction of Option B+ in Malawi have shown a sharp increase of ART initiations amongst pregnant HIV-positive women, which has been acknowledged as a success of the national PMTCT programme [2,3]. Full integration of PMTCT services in ANC clinics may offer the best opportunity of achieving high ART uptake due to direct linkage of care [9]. However, the model of integration in high prevalence, low resource contexts may also have an impact on downstream retention. Our current analysis confirms the finding from an earlier health facility-level analysis in an operational setting, where we observed that model of care had impact on retention in care [6], with worse program indicators in facilities in which women receive the first dose of ART at the ANC clinic and are then transferred to the ART clinic for ART follow-up.

Importantly, with patient-level data we are now able to demonstrate that same day HIV diagnosis and ART initiation is associated with increased risk of defaulting independently from the model of care. The potential negative impact of same day HIV diagnosis and ART initiation in an operational setting has been described before in a study that did not consider model of care [3] and may have many reasons [10]. Antenatal women may experience considerable peer and/or staff pressure to initiate ART. But if they are referred from ANC to an ART clinic, this may create a non-confrontational opportunity to defer or avoid ART initiation if they have misgivings, need time to disclose to their partners or require more reflection on the implications of their HIV diagnosis [11,12]. Retention on ART may be improved through improved counselling by health care staff at ANC clinics, by avoiding initiation of ART on the same day as HIV testing and/or by peer support at health facilities and in the community. Some of these interventions are currently being studied [13,14]. Our findings are compatible with the assumption that women need time to adjust to their HIV diagnosis before initiation on ART, no matter where it is offered. The challenge of making optimal use of the direct linkage of care in an integrated ANC-PMTCT service whilst respecting the individual's need for unhurried ART initiation is considerable in circumstances where many women visit an ANC clinic only once during pregnancy and often at a late stage of gestation [15].

This operational research has utilized routine MOH data to provide insight into optimal implementation of Option B+ in a real-world setting. Some care needs to be taken with the interpretation of our findings. Although we checked names and registration numbers of pregnant women across the study clinics, it is theoretically possible that women self-transferred to start ART at other health facilities. Results may not be representative of health facilities outside Zomba District or of larger, urban clinics, and we did not explore in great detail the effects of health systems, health care worker and patient-level factors on retention in care. A further limitation is that we did not study the reasons why health facilities chose the model that they implemented and how that choice might have affected the outcomes that were observed

## Conclusions

In health facilities where ANC clinics provide integrated HTC and ART initiation services (Model 1), we observed significantly higher uptake of ART but lower retention on ART, compared with health facilities where ANC clinics only provide HTC and ART was initiated outside the ANC clinic (Model 2). Initiation of ART on the same day as HIV diagnosis was associated with reduced retention in the first six months after ART initiation, independent of model of care. Further context-specific, patient-level research is needed to evaluate how retention may be impacted on by a same-day “test and treat” strategy in high prevalence, low resource settings. Such research would have important implications beyond the prevention of vertical transmission, given the interest in adopting “test and treat” for all people living with HIV by the WHO in the near future.
